# Efficacy and safety assessment of homotopical transplantation of iPSCs‐derived midbrain organoids into the substantia nigra of Parkinsonian rats

**DOI:** 10.1002/btm2.70014

**Published:** 2025-03-27

**Authors:** Xin Zheng, Jianwei Chen, Zhengzheng Huang, Youcheng Zhang, Liping Zhou

**Affiliations:** ^1^ Department of Neurology The First Affiliated Hospital of Shenzhen University, Shenzhen Second People's Hospital Shenzhen China; ^2^ Department of Neurosurgery, Shenzhen Key Laboratory of Neurosurgery The First Affiliated Hospital of Shenzhen University, Shenzhen Second People's Hospital Shenzhen China; ^3^ Institute of Pharmacy and Molecular Biotechnology (IPMB) & BioQuant Universität Heidelberg Heidelberg Germany; ^4^ School of Optometry The Hong Kong Polytechnic University Kowloon Hong Kong; ^5^ Department of Applied Biology and Chemical Technology The Hong Kong Polytechnic University Kowloon Hong Kong

**Keywords:** homotopical transplantation, midbrain organoid, nigrostriatal pathway reconstruction, Parkinson's disease

## Abstract

Current ectopic implantation has shown limited efficacy in promoting reinnervation of the nigrostriatal pathway, which is critically affected in Parkinson's disease (PD). Homotopic transplantation, on the other hand, may facilitate physiological cell rewiring of the basal ganglia, potentially improving PD symptoms. This study aimed to evaluate the efficacy and safety of homotopically engrafting human induced pluripotent stem cells (hiPSCs)‐derived midbrain organoids into the substantia nigra of PD rats. A rat model of PD was induced using 6‐hydroxydopamine (6‐OHDA) and homotopically transplanted into the lesioned SN with hiPSC‐derived hMOs. The engrafted hMOs survived and continually mature in host brains, and were mainly differentiated into dopaminergic lineage neurons, part of which presented TH^+^ fibers. Behavioral evaluation demonstrated that transplantation of hMOs gradually reverse the motor disorder caused by 6‐OHDA lesioning by 22% at week 5 and 35% by week 10 post‐transplantation, respectively. No tumor formation or migration was detected in either subcutaneous space or vital organs following 10 weeks implantation. These findings support the efficacy and safety of homotopical hMOs transplantation, offering a promising cell‐based strategy for treating Parkinson's disease.

**Abbreviations**
2‐D: 2‐dimentional
6‐OHDA: 6‐hydroxydopamine
APO: apomorphine
BDNF: brain‐derived neurotrophic factor
cAMP: dibutyryladenosine 3′,5′‐cyclic monophosphate sodium salt
DA: dopaminergic
DAPT: 1‐dimethylethyl ester
DIV: days in vitro
EGFP: enhanced green fluorescent protein
EN1: engrail‐1
FGF8: fibroblast growth factor 8
FOXA2: forkhead box A2
fVM: Fetal ventral mesencephalon
GDNF: glial cell‐derived neurotrophic factor
GIRK2: G‐protein‐coupled inward rectifier potassium
hiPSCs: human induced pluripotent stem cells
hMOs: human midbrain organoids
hNA: human cell nuclei
hNCAM: human neural cell adhesion molecule
HPLC: high‐performance liquid chromatography
NURR1: nuclear receptor subfamily 4
OTX2: orthodenticle homeobox 2
PD: Parkinson's disease
SHH: Sonic hedgehog
SN: substantia nigra
SOX2: sry‐box transcription factor 2
TH: tyrosine hydroxylase
TUJ1: tubulin beta 3 class III

**Translational Impact Statement**

Homotopically transplanted human iPSC‐derived midbrain organoids (hMOs) successfully survive and retain their dopaminergic lineage identity in the substantia nigra of rats with 6‐OHDA‐induced Parkinson's disease. The transplanted hMOs significantly enhance the behavioral performance of PD rats without causing tumorigenicity in vital organs, indicating their potential efficacy and safety for Parkinson's disease treatment.

## 1 INTRODUCTION

Parkinson's disease (PD) is the second most common neurodegenerative disorder, characterized by the progressive loss of dopaminergic neurons (DA) in the substantia nigra pars compacta (SNpc).^1^ This neuronal loss disrupts nigrostriatal neurotransmission, leading to diminished dopamine release in the striatal region and resulting in motor dysfunction and other debilitating symptoms.^2^ Cell transplantation has emerged as a promising therapeutic strategy to replenish lost DA neurons, aiming to enhance DA neurotransmitter release and facilitate the integration of grafts into host neural circuit.^3^ Initial efforts in cell transplantation focused on fetal ventral mesencephalic (fVM) tissue.^4^, ^5^, ^6^, ^7^, ^8^, ^9^, ^10^, ^11^, ^12^, ^13^, ^14^ However, ethical, along with technical challenges and immune rejection, limit the widespread application of this approach.^15^ Therefore, there is a critical need for effective methods to obtain dopaminergic neurons while minimizing immune responses.^15^

The development of human induced pluripotent stem cells (hiPSCs) has revolutionized cell‐based therapy by enabling the reprogramming of somatic cells into pluripotent cells that can differentiate into various cell types, including dopaminergic progenitors.^16^, ^17^ This autologous approach reduces the risk of immune rejection and circumvents ethical issues associated with fetal tissue procurement. Recent studies have shown that iPSC‐derived dopaminergic neurons can improve motor symptoms in preclinical models and clinical trials.^13^, ^15^, ^18^, ^19^, ^20^, ^21^, ^22^ Despite these advances, traditional two‐dimensional (2D) culture systems do not adequately mimic the complex architecture and cellular interactions found in native tissues. The emergence of three‐dimensional (3D) organoids has addressed these limitations by promoting cell–cell and cell‐extracellular matrix interactions that are essential for proper neuronal function.^23^, ^24^ Previous work from our team demonstrated that ectopically transplanted human midbrain organoids (hMOs) could reverse motor deficits in a PD mouse model without causing tumor formation or graft overgrowth.

Ectopic and homotopic transplantation are two distinct approaches used in regenerative medicine and transplantation research. Ectopic transplantation involves placing cells or tissues in a non‐native location, which can facilitate easier monitoring and access but may not fully replicate the natural microenvironment. In contrast, homotopic transplantation places cells or tissues in their original anatomical location, potentially offering a more physiologically relevant environment that supports natural integration and function. Meanwhile, it lacks the detailed insights into how the local microenvironment influences the integration and function of transplanted tissues. Regarding the cell transplantation therapy for PD treatment, grafts can be implanted either homotopically into the substantia nigra (SN) region or ectopically in the striatum (STR) region.^22^ While ectopic transplantation has been favored due to shorter axonal travel distances to target regions, it fails to achieve complete reconstruction of the nigrostriatal circuit and does not allow for proper functional regulation by host neurons.^20^, ^25^, ^26^ In contrast, homotopic transplantation into the substantia nigra region may facilitate more accurate neural reconstruction and synaptic input regulation from host neurons.^25^, ^27^

Findings from our preliminary study indicate that hMOs transplanted homotopically into the SN region resulted in a prolonged improvement in motor function compared to ectopic striatum transplantation. This is consistent with previous studies showing superior outcomes for substantia nigra transplants in behavioral assessments.^22^ Given these insights, this study aims to evaluate the efficacy and safety of homotopically engrafting iPSC‐derived midbrain organoids into the substantia nigra of PD rats. We hypothesize that homotopic organoid transplantation will enhance therapeutic outcomes by promoting effective reconstruction of the nigrostriatal circuit, while also ensuring the safety and viability of the transplanted organoids.

## 2 RESULTS

### 2.1 Differentiation of hiPSCs to hMOs in a 3D culture manner

**FIGURE 1:**
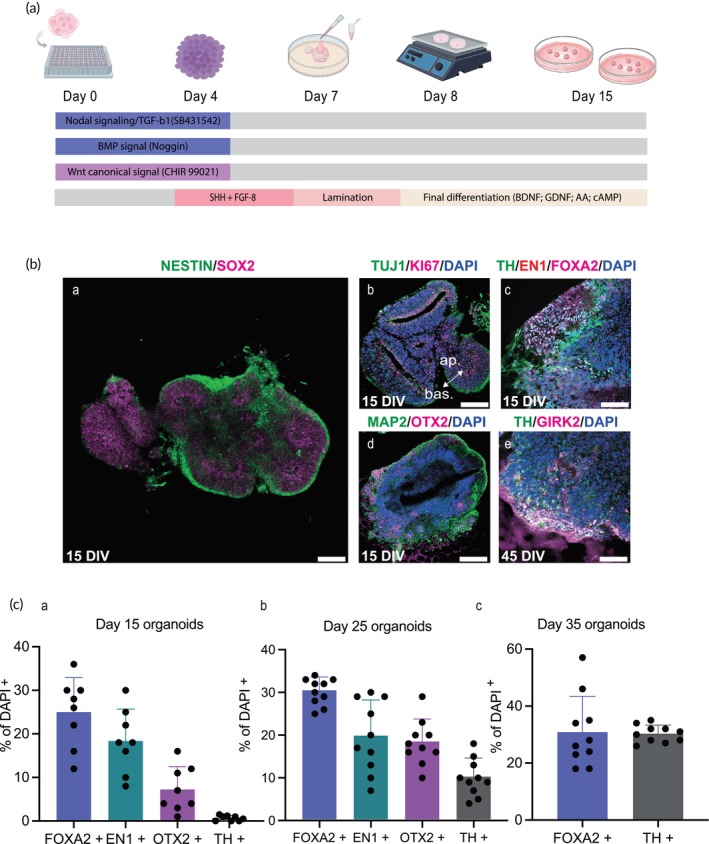
Differentiation of human induced pluripotent stem cells (hiPSCs) to hMOs. (a) Schematic representation of the differentiation process from hiPSCs to hMOs. (b) Immunofluorescent staining characterizing hiPSC‐derived hMOs (*n* = 10). Scale bars: 100 μm. (c) Proportions of cells positive for specific dopaminergic neuron‐related markers were calculated by dividing the cell number labeled with them by the total cell number labeled with DAPI at days 15, 25, and 35, respectively (*n* = 10). FGF8, fibroblast growth factor 8; SHH, Sonic hedgehog; BDNF, brain‐derived neurotrophic factor; GDNF, glial cell‐derived neurotrophic factor; cAMP, dibutyryladenosine 3′,5′‐cyclic monophosphate sodium salt; AA, Ascorbic acid; SOX2, sry‐box transcription factor 2; TH, tyrosine hydroxylase; TUJ1, tubulin beta 3 class III; MAP2, microtubule‐associated protein 2; OTX2, orthodenticle homeobox 2; FOXA2, forkhead box A2; EN1, engrail‐1; NURR1, nuclear receptor subfamily 4; GIRK2, G‐protein‐coupled inward rectifier potassium; DIV, days in vitro; ap., apical; bas., basal.

The hiPSCs were differentiated into hMOs using a previously described protocol^28^, ^29^ (Figure 1A). hiPSCs were cultured on Matrigel until confluent, then dissociated and seeded in a low‐adhesion 96‐well plate to initiate differentiation (Figure S1A,B). By day 4, embryonic bodies had formed. Neural rosettes were first identified at day 7 and continued to expand until day 15 (Figure S1B). At this stage, the organoids were sufficiently large for immunofluorescence staining (Figure 1B). Cells expressed early neuronal markers (NESTIN and SOX2) and early dopaminergic progenitor markers (FOXA2, EN1, and OTX2), indicating directed differentiation towards the midbrain DA lineage (Figure 1B, a,c,d). The proliferation marker KI67 was predominantly expressed compared to mature neuronal markers TUJ1 and MAP2, with KI67 and TUJ1 localized at apical and basal regions, respectively (Figure 1B, b). The immunofluorescence staining results revealed that day 15 hMOs composed approximately 29.5% FOXA2^+^, 18.25% EN1^+^, and 9.56% OTX2^+^ cells, with a small fraction (0.79%) positive for the mature DA marker TH (Figure 1C, a). By day 25, the proportions of FOXA2^+^, EN1^+^, OTX2^+^, and TH^+^ cells in hMOs were 30.75%, 20.25%, 14.44%, and 9.67%, respectively (Figure 1C, b). At day 35 in vitro, FOXA2^+^ and TH^+^ cells increased to 32.67% and 32.25%, respectively (Figure 1C, c).

The organoids were subsequently transferred to 6‐well plates and placed on an orbital shaker for continued maturation. During this period, the organoids increased in size and developed axons to form a dense neuronal network. The midbrain A9 region‐specific marker GIRK2 was detected until day 45 (Figure 1B, e), confirming the successful generation of human midbrain organoids (hMOs). To evaluate the functional activity of the neuronal network, a multi‐electrode array (MEA) system was employed to record extracellular field potentials.^30^, ^31^, ^32^ Synchronous network‐wide bursts were identified in the hMOs, indicating the development of interconnections and electrophysiological activity (Figure S2).

### 2.2 Engraftable hiPSC‐derived hMOs were homotopically transplanted into the substantia nigra (SN) of PD rats

Before assessing in vivo efficacy, PD animals were established, and motor function was evaluated in PD rats prior to and following transplantation. Rats were selected for this study due to their larger substantia nigra, which facilitates intracranial stereotactic injections compared to mice. Parkinsonian rats were generated by injecting 6‐OHDA into the medial forebrain bundle (MFB) using stereotactic techniques. Four weeks post‐lesioning, the success of the 6‐OHDA rat model was confirmed using the Apomorphine (APO) induced rotation test. Rats exhibiting more than 100 turns in 30 min were selected for transplantation.

**FIGURE 2:**
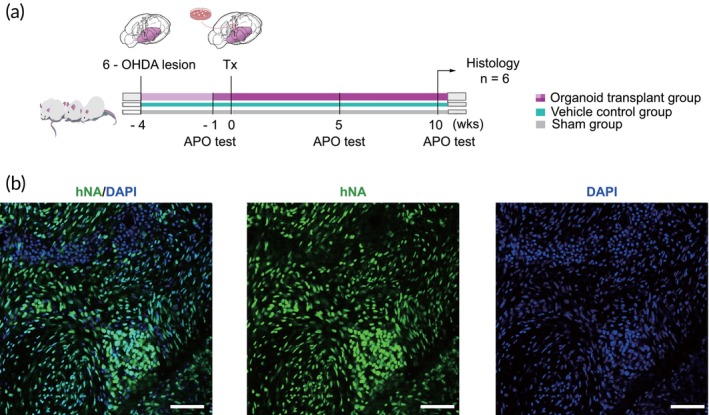
Transplantation of hiPSC‐derived hMOs into PD rats. (a) Schematic representation of the experimental design and timeline of behavioral tests and histology analysis. (b) Engrafted cells were identified by immunohistochemical staining (IHC) of hNA and hNCAM. (c) Representative confocal microscopic images confirmed the survival of transplanted cells. Scale bars: 100 μm.

Day 15 hMOs were then transplanted into the SN of unilaterally 6‐OHDA‐lesioned PD rats (Figure 2a). Behavioral assessments were conducted at 5 and 10 weeks post‐transplantation. At the end of the study (10 weeks), rats were sacrificed for histological analysis. Numerous survival cells were identified through immunofluorescence staining using a human‐specific nuclei antibody (hNA) and a neural cell adhesion molecule antibody (hNCAM) (Figure 2b,c).

### 2.3 Engrafted hMOs maintained dopaminergic lineage identity in vivo

**FIGURE 3:**
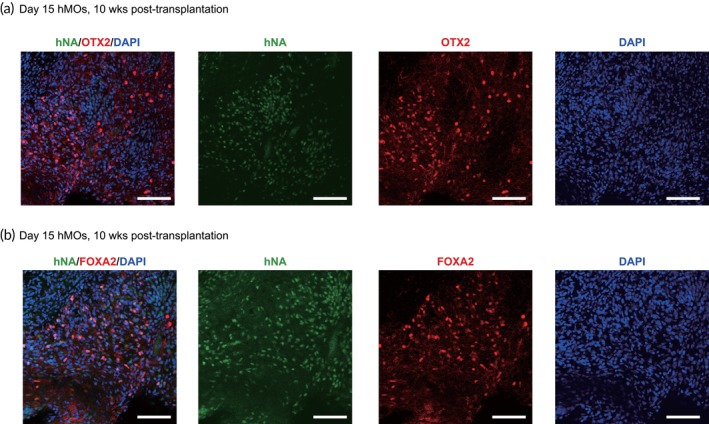
The hiPSC‐derived hMOs maintained dopaminergic neuron identities in vivo. (a, b) Representative confocal microscopic images showed that engrafted hMOs still maintain the properties of dopaminergic lineage. Scale bars, 100 μm.

To determine whether the engrafted hMOs retained their dopaminergic lineage identity after the transition from in vitro to in vivo conditions, immunofluorescence staining was performed to characterize the identity of the engrafted cells. The results confirmed that the majority of engrafted cells expressed high levels of the dopaminergic specific progenitor markers OTX2 (Figure 3a) and FOXA2 (Figure 3b), indicating that the engrafted cells remained committed to the dopaminergic lineage cells.

### 2.4 Transplantation of hiPSC‐derived hMOs eventually differentiated into DA lineage neurons in the SN of PD rats

**FIGURE 4:**
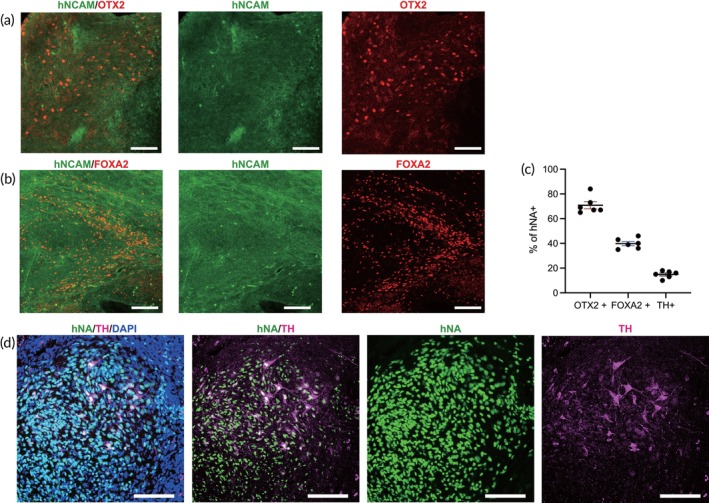
Phenotype of terminally differentiated DA neurons of hMOs in vivo. (a, b) Representative confocal microscopic images showed that considerable engrafted cells expressed dopaminergic progenitor markers OTX2 or FOXA2, co‐staining with the axonal fiber marker hNCAM. Scale bars, 100 μm. (c) 70.83% of hNA^+^ stained positive for OTX2, 39.83% of hNA stained positive for FOXA2, and 14.83% of hNA^+^ stained positive for TH. (d) Grafted‐derived TH^+^ axons were visualized 10 weeks post‐transplantation. Scale bars, 100 μm.

Following transplantation, the engrafted hMOs were required to continually differentiate into mature DA in vivo. This was verified through immunofluorescence staining using hNCAM, a marker specific for neural cells and their fibers. The results showed that nearly all grafted neurons labeled with hNCAM were also positive for dopaminergic progenitor markers OTX2 (70.83% of hNA^+^) and FOXA2 (39.83% of hNA^+^) (Figure 4a–c), indicating that most engrafted cells differentiated into dopaminergic lineage cells with extensive axonal extension. At 10 weeks post‐transplantation, 14.83% of hNA^+^ cells were positively stained for TH, a marker of mature dopaminergic neurons. These findings suggest that while the majority of the engrafted hMOs differentiated into dopaminergic lineage cells, as indicated by high expression of FOXA2 and OTX2, only a subset progressed to full maturation into TH^+^ dopaminergic neurons within 10 weeks post‐transplantation.

The complete hemi‐sided lesioning of rat DA neurons was confirmed by the loss of TH staining in the striatum, MFB, and substantia nigra (Figure S3), correlating with APO‐induced rotational asymmetry. After 10 weeks of hMOs transplantation, a small proportion of engrafted cells (hNA^+^) matured over time and presented graft‐derived TH^+^ fibers (Figure 4d).

### 2.5 Cell compositions of transplanted hMOs in vivo

**FIGURE 5:**
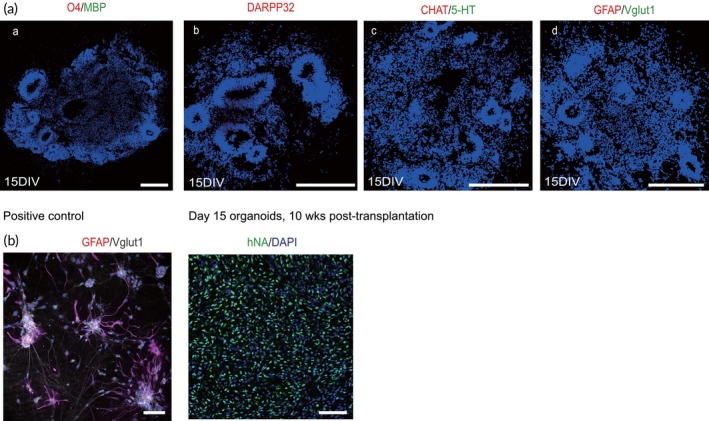
Cell compositions of Transplanted hMOs in vitro and in vivo. (a) Representative confocal microscopic images showed that day 15 hMOs did not contain other types of cell lineage markers including O4 and MBP for oligodendrocytes, DARPP32 for medial spiny neurons, CHAT for cholinergic, SEROTONIN for serotonergic neurons, GFAP for astrocytes, and Vglut1 for glutamatergic (*n* = 10). Scale bars, 100 μm. (b) Representative confocal microscopic images demonstrated that engrafted hMOs were not differentiated into astrocytes or glutamatergic neurons (*n* = 8). Scale bars, 100 μm.

To determine whether engrafted hMOs change their cell identities and differentiate into non‐dopaminergic neurons or glial cells, immunofluorescence staining was performed on both in vitro and in vivo samples. As shown in Figure 5, no graft‐derived glial cells (such as astrocyte and oligodendrocyte) or other neuronal populations (including glutamatergic neurons, cholinergic neuron, serotonergic neurons and medial spiny neurons) were detected in either the day 15 hMOs in vitro (Figure 5a) or the in vivo grafted cells (Figure 5b).

### 2.6 Transplantation of hMOs restored movement disorders in PD rats

**FIGURE 6:**
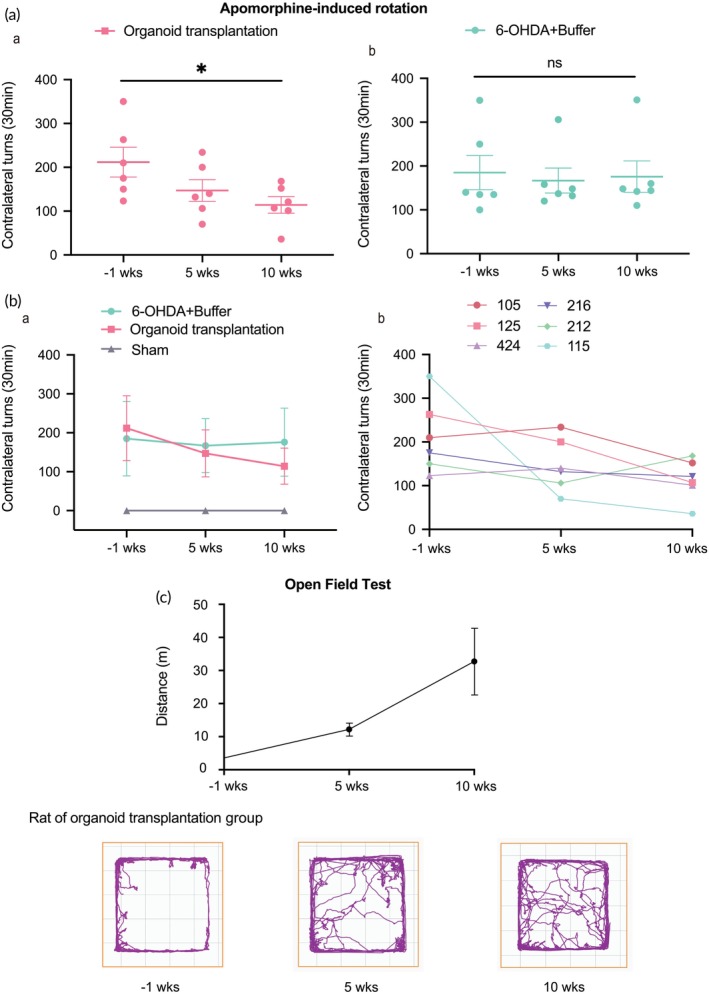
Restoring effects of hMOs grafts on motor function. (a) Results of APO‐induced rotation in the group with hMOs transplantation (a) and the group without hMOs transplantation (b). *n =* 6, *p < 0.05 by one‐way ANOVA. (b) Results of APO‐induced rotation in three groups of rats (a). Results of APO‐induced rotation in individual rats from the organoids transplantation group (b). (c) Results of the open field test in the group with hMOs transplantation and representative rat moving traces in the open field experiment.

To examine the efficacy of hMOs grafts in restoring motor functions, APO‐induced rotation was conducted at 5 and 10 weeks post‐transplantation. PD rats that received hMO grafts displayed significant improvement in asymmetric movement over the 10‐week treatment period (Figure 6A, a), while untreated PD rats showed no improvement (Figure 6A, b). Interestingly, the functional recovery of motor function started to be observed at 5 weeks post grafting. The results demonstrated that hMOs transplantation can gradually reverse the motor deficits caused by 6‐OHDA lesioning (Figure 6B). In addition, the treatment group demonstrated enhanced motor performance in the open field test (Figure 6C).

### 2.7 Tumorigenicity study of transplanted hMOs

**FIGURE 7:**
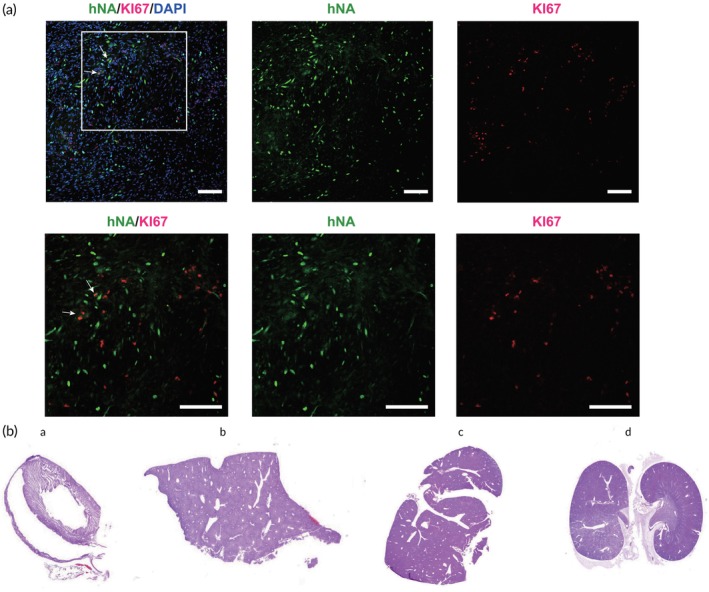
Tumorigenicity study of transplanted hMOs in PD rats. (a) The grafted sites were negative for the KI67 and OCT4 staining. Scale bars, 100 μm. (b) None of the tumor migration was detected in the subcutaneous space or in organs, such as the heart (a), liver (b), spleen (c), and kidney (d). Scale bars, (a–d), 1 mm.

A critical concern for hiPSC‐based cell therapy is ensuring safety by preventing teratoma formation due to contamination with pluripotent stem cells. At 10 weeks post‐transplantation, engrafted organoids were negative for OCT4 and KI67, indicating a low tumorigenic risk (Figure 7A). Additionally, day 15 organoids were injected subcutaneously into severe combined immunodeficient (SCID) mice, along with Matrigel, as a sensitive method for detecting teratoma formation. No tumor formation or migration was detected in the subcutaneous space or in vital organs, including the kidney, liver, heart, and spleen (Figure 7B, a–d).

## 3 DISCUSSION

Parkinson's disease is characterized by the loss of DA neurons in the substantia nigra compacta (SNpc) region.^33^ Cell transplantation is considered a viable approach for replenishing lost DA neurons.^3^ A critical factor influencing the efficacy of clinical neural transplantation for PD is the placement of the graft. Current strategies that primarily focus on ectopic grafting in the striatum (ST) fail to adequately reconstruct the basal ganglia circuitry or normalize neuronal activity in key structures, such as the SNPc and subthalamic nucleus (STN).^34^ Previous studies have employed a “double grafting” strategy, targeting both the ST and SNPc with dopaminergic grafts,^35^, ^36^ and demonstrated improved recovery of complex sensorimotor behaviors in the PD rat model.^36^ The present study is the first to show that midbrain organoids transplanted into the SNPc region can survive and differentiate into DA lineage neurons without causing aberrant overgrowth, indicating both the safety and efficacy of this treatment.

A significant challenge in SNPc transplantation is ensuring cell survival,^37^, ^38^ as apoptotic neurons can be observed in grafts as early as 24 h post‐implantation. Surviving dopaminergic neurons are typically localized at the periphery of the transplants,^19^ likely due to insufficient trophic support^39^, ^40^ and poor vascularization.^41^ Vascularization is crucial for brain organoids, as it enhances oxygen penetration, nutrient supply, and efficient neural progenitor differentiation.^42^ However, the SNPc region is less vascularized compared to the blood‐rich striatum, leading to necrosis in the center of the organoid, which can hinder normal development and neuronal migration.^43^ To enhance graft survival, we utilized small organoid pieces for transplantation, increasing contact with host tissue and improving nutrient distribution.^39^

The therapeutic efficacy of grafts, assessed by the restoration of motor function, correlates with the ability of grafted midbrain DA neurons to establish a new terminal network with the host striatum.^44^ Early studies using fetal tissue grafts or mDA neurons generated from human pluripotent stem cells have shown that while DA neurons can extend axons to the striatum, the level of connectivity achieved is often insufficient to restore motor function effectively.^27^ Our findings indicate that homotopic grafts alone did not provide adequate striatal innervation to fully reverse motor deficits induced by 6‐OHDA lesions, consistent with recent studies on pluripotent stem‐cell‐derived DA grafts in similar 6‐OHDA lesioned rats.^25^ Conversely, another study demonstrated that homotopic grafts can achieve impressive levels of target‐specific pathway reconstruction, suggesting that differences in species (mouse vs. rat) and associated anatomical scales may significantly influence outcomes.^22^ This highlights the challenge of long‐distance axonal growth in the adult brain, emphasizing the need for scalable strategies in human applications.

The choice between homotopical and ectopic transplantation strategies presents distinct advantages and challenges. Homotopical transplantation, as demonstrated in our study, allows for the direct integration of grafts into the SNpc, facilitating more accurate reconstruction of the nigrostriatal circuit and better functional regulation by host neurons. In contrast, ectopic transplantation into the striatum, while easier to perform and associated with shorter axonal travel distances, may not achieve the same level of circuit reconstruction or functional recovery. Studies have shown that ectopic grafts often fail to establish proper synaptic connections with host neurons, leading to suboptimal functional outcomes.^45^ Moreover, the risk of graft overgrowth and tumor formation may be higher in ectopic placements due to the less controlled environment and the potential for increased cell proliferation.^46^ Overall, while both strategies have their merits, homotopical transplantation may offer enhanced safety and efficacy by promoting closer anatomical and functional integration with host neural circuits.

Research into specific neuronal guidance molecules has revealed that cell‐derived neurotrophic factors can promote axon extension from the SNpc to the striatum, facilitating long‐distance directional axon growth in the host brain.^47^ Overexpression of glial cell‐derived neurotrophic factor (GDNF) in the striatum can induce substantial axons to extend long distances from the substantia nigra to the striatum, yielding improvement in motor function comparable to those achieved with conventional ectopic transplantation.^25^ Additionally, Netrin‐1 has been shown to play a role in directing the projection of neuronal axons.^48^ Our group has observed directed axonal elongation of transplanted organoids following GDNF or Netrin‐1 overexpression in the nigrostriatal circuit (data not shown). While cell‐intrinsic factors regulate the ability of graft fibers to innervate target structures, synaptic integration is largely determined by graft placement, as shown in studies involving hESC‐derived GABAergic transplants.^20^, ^22^ Future studies should focus on optimizing axonal extension and synaptic integration of transplanted organoids to enhance outcomes.

Studies have indicated that at 8 weeks post‐transplantation, TH^+^ cells make up approximately 8% to 10% of the total transplanted cell population, while dopaminergic progenitor cells expressing FOXA2 account for 80% of the total transplanted cells.^49^ In our study, the proportion of mature dopaminergic neurons was approximately 14%, which is consistent with these previous findings. FOXA2 and OTX2 are transcription factors that play crucial roles in the early differentiation and specification of dopaminergic progenitors. Their high expression levels in the transplanted hMOs suggest that a significant number of engrafted cells are in the progenitor stage, actively differentiating along the dopaminergic lineage. However, the transition from progenitor to mature dopaminergic neurons requires additional time and specific environmental cues. TH^+^ cells become detectable around 6 weeks post‐transplantation; however, complete neuronal maturation requires more than 16 weeks of graft development.^29^, ^49^, ^50^ This likely contributes to the observed discrepancy, where there is a high proportion of FOXA2^+^ and OTX2^+^ neurons compared to a relatively low proportion of TH^+^ neurons. While many cells have committed to the dopaminergic lineage (FOXA2^+^ and OTX2^+^ neurons), they may still be undergoing maturation processes necessary to fully express TH and acquire functional characteristics of mature dopaminergic neurons.^51^ Even though previous studies demonstrate that 500–700 surviving fVM DA neurons are sufficient to facilitate motor function recovery in an animal model.^52^, ^53^ Therefore, despite the relatively low number of mature dopaminergic neurons observed in vivo, functional recovery remains achievable, as demonstrated in the current study. However, direct evidence of functional synaptic integration has not yet been established and requires further investigation.

There are some limitations in the present study. In this study, we exclusively used male cell lines and animal models, which reflect the higher incidence of PD observed in males. According to epidemiological data, the incidence of PD is significantly higher in males compared to females, with twice the prevalence among males than females in any specific time frame suffering from PD. This sex‐specific difference is crucial for understanding the clinical relevance of our findings. However, it is important to acknowledge that using only one sex may limit the generalizability of our results to the entire population. Future studies should consider including both sexes to fully elucidate the potential sex‐based differences in PD pathophysiology and treatment response. Besides, the observation period for tumorigenicity at a maximum 10 weeks post‐transplantation is too short, and a longer observation period should be performed in future studies regarding the safety evaluation of homotopic organoid transplantation.

## 4 MATERIALS AND METHODS

### 4.1 Cells culture and generation of hMOs

hiPSCs (male) were cultured on a feeder‐free Matrigel surface in E8‐mTESR medium (STEMCELL™, Vancouver, Canada; catalog # 05990). The protocol for hMOs induction was based on previous work by Jo and Nolbrant S, with minor modification.^28^, ^29^ In brief, hiPSCs were dissociated into single cells using TrypLE™ Express (Gibco, Waltham, MA, USA; 12563029), then seeded in a low‐cell‐adhesion 96‐well plate at a density of 10,000 cells per well (Corning, Corning, NY, USA) to form embryoid bodies. The wells were filled with a neuronal induction medium composed of DMEM/F12™ (Gibco, 10565‐018) and Neurobasal™ (Gibco, 21103049) at a 1:1 ratio, supplemented with 1:100 N2™ supplement (Gibco, 17502048), 1:50 B27™ without vitamin A (Gibco, 12587010), 1% minimal essential media‐nonessential amino acid (Gibco, 11130051), and 0.1% β‐mercaptoethanol (Invitrogen, Waltham, MA, USA; 31350010). The medium also contained 10 μM SB431542 (Stemgent, Cambridge, MA, USA), 100 ng/mL Noggin (Peprotech, Rocky Hill, NJ, USA), 0.8 μM CHIR99021 (Selleck, S2924), SHH (300 ng/mL R&D system; 200 ng/mL Peprotech) and 10 μM ROCK inhibitor Y27632 (Calbiochem, San Diego, CA, USA). The ROCK inhibitor was applied for the first 48 h, with medium changes every 2 days. On day 4, the supplements were replaced with 100 ng/mL FGF8 (Peprotech) for midbrain patterning. After an additional 4 days, the hMOs were cultured with 20 ng/mL BDNF (Peprotech), 10 ng/mL glial cell line‐derived neurotrophic factor (GDNF) (Peprotech), 200 μM ascorbic acid (Sigma‐Aldrich, St. Louis, MO, USA), and 500 μM db‐cAMP (Sigma‐Aldrich). Cultures were maintained on an orbital shaker (set at 70 rpm) to facilitate nutrient and oxygen exchange with medium replacement every 3 days. The induction of the iPSC‐derived DA neurons in 2D culture was conducted following the same growth factors regimen used for hMOs induction.

### 4.2 PD model establishment and transplantation of hMOs

All animal procedures were performed in accordance with the 3R principle and were approved by the ethics committee of The First Affiliated hospital of Shenzhen University (Animal protocol number: 20240084). A total of 80 Sprague–Dawley (SD) rats (8–12 weeks old, Charles River, Beijing, China) were used in this study, with 60 of them employed to establish a PD model as previously reported.^54^ In brief, 12 μg of 6‐OHDA (6 mg/mL in saline with 0.2% ascorbic acid) was injected directly into the medial forebrain bundle (coordinates: anterior–posterior [AP] = 4.4 mm, lateral [L] = −1.2 mm, vertical [V] = −7.8 mm). Four weeks after lesioning, rats that displayed more than 100 rotations in a 30‐min period following apomorphine administration were considered successfully modeled for PD and were selected for transplantation experiments. Of the twelve 6‐OHDA‐lesioned rats, half were assigned to the vehicle control group and received only the transplantation buffer. Sample size was determined by performing a power analysis using G*Power, specifying an effect size of 0.7071068 calculated based on our preliminary observation, an alpha level of 0.05, and a desired power of 0.9. In compliance with the ARRIVE guidelines (https://arriveguidelines.org/arrive-guidelines), we implemented a rigorous randomization process to assign animals to experimental groups, thereby minimizing bias and enhancing the reliability of our findings. The animals were assigned individual numbers, which were maintained on ear tags until the sacrifice of the animals at the end of the study. We used a computer‐generated randomization schedule, which was created by performing SAS® Programming. This schedule ensured that each animal had an equal chance of being assigned to any of the experimental groups. The randomization process was conducted by an independent researcher who was not involved in the experimental procedures or data analysis, ensuring objectivity. By adhering to this method, we aimed to achieve comparable groups at the start of the experiment, thus enhancing the validity and reproducibility of our results. The remaining PD rats were assigned to the hMOs transplantation group. Before transplantation, day 13 organoids were treated with 40 μM quercetin for 16 h to remove any residual hiPSCs. The organoids were then digested and counted to determine the cell number, with three replicates per organoid. Organoids containing 4 × 10^5^ cells were divided into small fragments, ensuring each fragment could pass through a 10 μL pipette tip without obstruction. Transplantable organoids were resuspended in 4 μL transplantation buffer containing 0.5 μM Rock inhibitor, B27 without vitamin A, and 20 ng/mL BDNF and were subsequently injected into the right substantia nigra (coordinates: [AP] = 5.3 mm, lateral [L] = −2.0 mm, vertical [V] = −8.2 mm) using a microinjector (HAMILTON, SYR 1701 N, needle size 26 s G; Figure S1C). The injection rate was set at 0.5 μL/min, with the needle left in place for an additional 5 min before being slowly withdrawn.

### 4.3 Tissue processing and immunofluorescent staining

Rats were transcardially perfused with 50 mL of 0.9% saline at room temperature (21°C), followed by 100 mL of ice‐cold 4% paraformaldehyde (PFA) in phosphate‐buffered saline (PBS). The brains were then extracted and fixed in 4% PFA for an additional 2 h, after which they were re‐suspended in 20% sucrose (in 0.1 M PB) overnight and subsequently transferred to 30% sucrose for dehydration. Twelve series of 40‐μm‐thick coronal sections were collected at −20°C. For immunofluorescence and immunohistochemical staining, tissues were incubated with 3% donkey serum for 2 h to block non‐specific binding. The primary antibodies used, along with their respective working dilutions, are listed in Table S1. Visualization was achieved through a peroxidase‐based reaction followed by diaminobenzidine (DAB) precipitation (Goat anti‐rabbit, ZSGB‐BIO, Beijing, China), or with Alexa 488, Alexa 555, or Alexa 647‐conjugated secondary antibodies (Jackson ImmunoResearch, West Grove, PA, USA).

### 4.4 MED64 multi‐electrode array and whole cell patch clamp recording

The electrophysiological activity of hMOs was recorded on day 60 using a multi‐electrode array (MEA) device. Each microelectrode in the MED64 Probe (P515A model) measured 50 μm × 50 μm, with a spacing of 150 μm. Whole‐cell patch‐clamp recordings were carried out to assess the electrophysiological properties of the engrafted EGFP+ organoids. These recordings were made on acute brain slices in an artificial cerebrospinal fluid (ACSF) bath solution containing 125 mM NaCl, 2.5 mM KCl, 2 mM CaCl_2_, 1.25 mM NaH2PO4, 1 mM MgSO4, 25 mM glucose, and 26 mM NaHCO3. The internal solution consisted of 143 mM KCl, 8 mM NaCl, 10 mM HEPES, 1 mM MgCl_2_, 2% biocytin, 2 mM Na‐ATP, and 0.4 mM Na‐GTP. EGFP+ cells were visualized using a fluorescence microscope.

### 4.5 Microscopy and quantifications

Bright‐field images were captured using a Leica microscope, while fluorescence images were obtained with a Leica DMI6000 confocal microscope. To determine the proportion of EN1^−^, FOXA2^−^, OTX2^−^, NURR1^−^, and TH‐expressing cells among the total DAPI‐labeled cells in vitro, the number of cells labeled with one certain biomarker was counted from at least 10 sampling areas from each of the three organoids using Image J software (National Institutes of Health, Bethesda, MD, USA). The proportion of this certain marker was calculated by dividing the cell number labeled with this marker by the total number labeled with DAPI. The graft within the brain slices was outlined, and confocal microscopy was employed to visualize the FOXA2^+^, OTX2^+^, and hNCAM^+^ signals. Image J was utilized to manually count cells labeled either singly or doubly. The ratio of FOXA2^+^ cells and OTX2^+^ cells was obtained by dividing the number of FOXA2^+^ cells and FOXA2^+^ cells by the total number of hNCAM^+^ cells, respectively.

### 4.6 Behavioral test

#### 4.6.1 Drug‐induced rotation test

Rotational behavior induced by apomorphine (APO) was evaluated prior to and at multiple time points following the transplantation of hMOs. Rotations were recorded starting 5 min after an intradermal injection of APO (10 mg/mL in saline, 0.5 mg/kg). The net number of rotations (contralateral minus ipsilateral) over a 30‐min period was analyzed for each subject.

#### 4.6.2 Open field test

Spontaneous locomotor activity was measured using an automated open field system to determine total distance traveled. Rats were habituated in the open field arena (25.4 cm × 25.4 cm) for 10 min before testing, followed by a 15‐min observation period. Each animal was assessed 1 week prior to organoid transplantation and re‐assessed at 5 and 10 weeks post‐transplantation. The total movement distance is presented in meters.

The behavioral assessment was conducted by the technician who was aware of the animal's individual number and not their group assignments. On the other hand, the data analysis was performed by another technician who knew the different groups but was unaware of the specific treatments. This approach was designed to minimize bias in the results due to human factors.

### 4.7 Statistical analysis

All data were presented as mean ± standard error of the mean (SEM). The Shapiro–Wilk test was performed to determine the normality of the data. The inter‐group differences were determined by one‐way ANOVA with Tukey's post hoc test (normal distribution) or Kruskal–Wallis test (non‐normal distribution) using GraphPad Prism 9. For behavioral test results, two‐way ANOVA followed by Bonferroni's multiple comparisons was employed. Statistical significance was set at a *p* value of <0.05.

## AUTHOR CONTRIBUTIONS

**Xin Zheng:** Methodology; investigation; funding acquisition; supervision; project administration. **Jianwei Chen:** Methodology; investigation. **Zhengzheng Huang:** Methodology. **Youcheng Zhang:** Formal analysis; writing – review and editing. **Liping Zhou:** Supervision; writing – review and editing; funding acquisition.

## CONFLICT OF INTEREST STATEMENT

The authors declare no conflicts of interest.

## DATA VISUALIZATION AND STATISTICAL ANALYSIS

All data points are to be shown in figures, including in column or bar plots to improve data transparency, and that the statistical analysis methods are fully described, justified, and the number of samples per experimental group is specified in the text.

## Supporting information


**Figure S1.** Differentiation of human induced pluripotent stem cells (hiPSCs) to hMOs. (A) Representative confocal microscopic images showed that iPS cells expressed pluripotent protein markers NANOG, SSEA3, OCT3/4, TRA‐1‐60. Scale bars, 100 m. (B) The morphology of organoids at different time points. (C) organoids preparation for transplantation.

**Figure S2.** Electrophysiological activity in intact organoids was evaluated using a MED64 multi‐electrode array system. (a) Diagram illustrating the electrophysiological signal processing setup. (b) A representative example of a spike cluster (marked by an arrow in panel c); (c) Synchronized bursts observed across multiple channels indicated the occurrence of a network burst (highlighted in the boxed region).

**Figure S3.** TH staining in the striatum, MFB, and substantia nigra of the 6‐OHDA rats. A. Cross section of the striatum, MFB and substantia nigra; B. TH‐positive neurons in the substantia nigra.

**Table S1.** Antibody information.

## Data Availability

The data that support the findings of this study are available on request from the corresponding author. The data are not publicly available due to privacy or ethical restrictions.
