# Supplementary figures and images for: Efficacy and safety assessment of homotopical transplantation of iPSCs‐derived midbrain organoids into the substantia nigra of Parkinsonian rats
Source: Bioeng Transl Med. 2025 Mar 27;10(5):e70014. doi: 10.1002/btm2.70014 (PMC12478334; doi:10.1002/btm2.70014)

**A**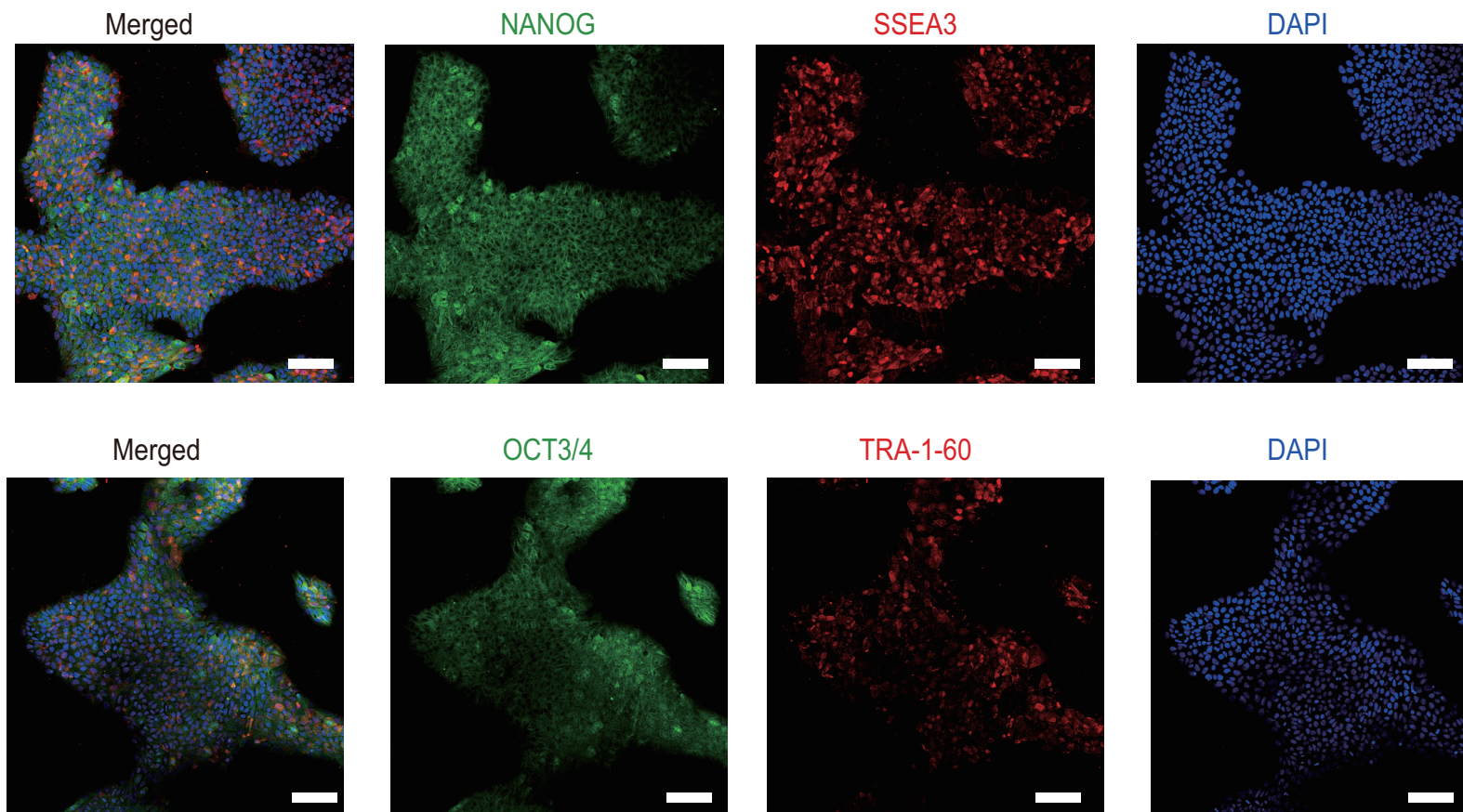**B**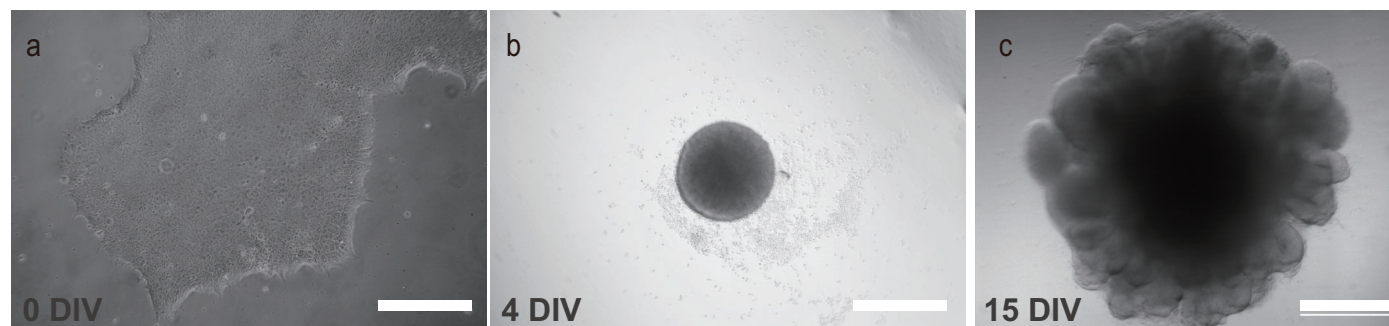**C**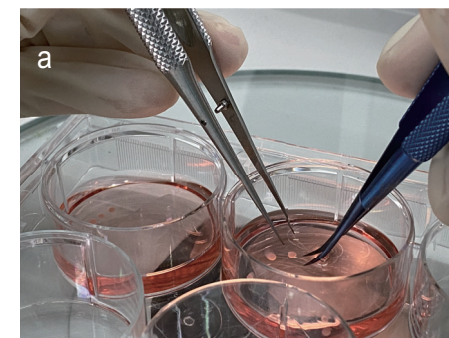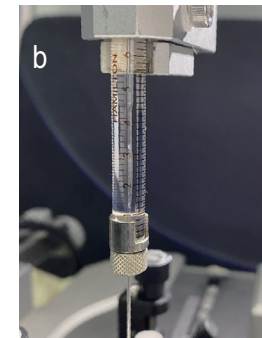

Supplement: Supplementary file 1 — Figure S1. Differentiation of human induced pluripotent stem cells (hiPSCs) to hMOs. (A) Representative confocal microscopic images showed that iPS cells expressed pluripotent protein markers NANOG, SSEA3, OCT3/4, TRA‐1‐60. Scale bars, 100 m. (B) The morphology of organoids at different time points. (C) organoids preparation for transplantation. [file BTM2-10-e70014-s001.pdf]

A

a

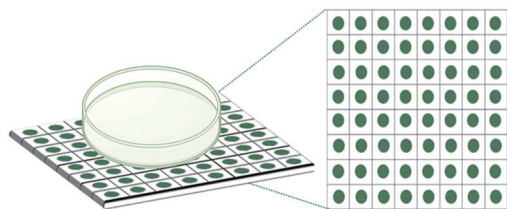

b

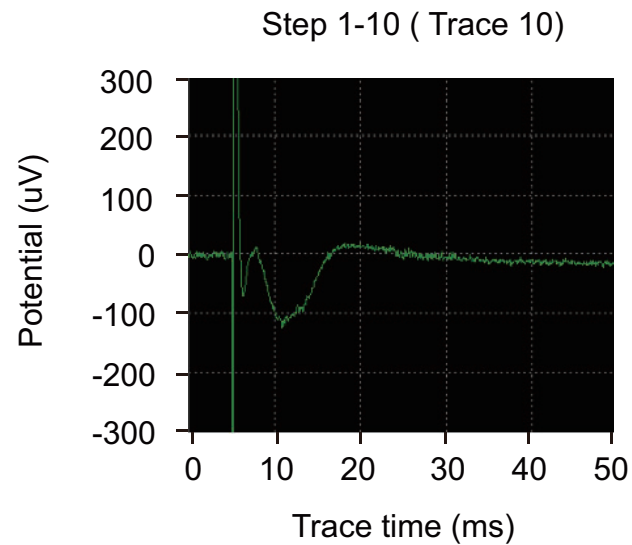

c

Overview of all channels

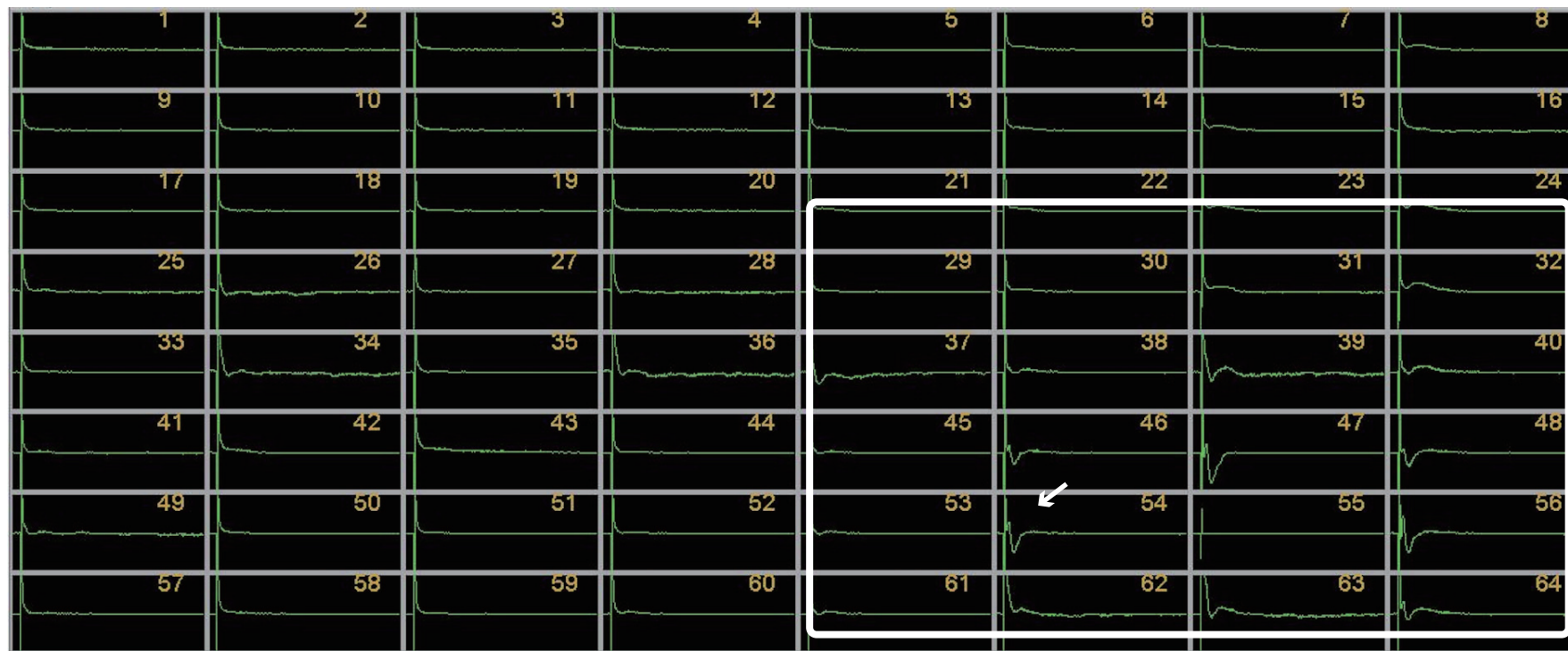

Supplement: Supplementary file 2 — Figure S2. Electrophysiological activity in intact organoids was evaluated using a MED64 multi‐electrode array system. (a) Diagram illustrating the electrophysiological signal processing setup. (b) A representative example of a spike cluster (marked by an arrow in panel c); (c) Synchronized bursts observed across multiple channels indicated the occurrence of a network burst (highlighted in the boxed region). [file BTM2-10-e70014-s004.pdf]

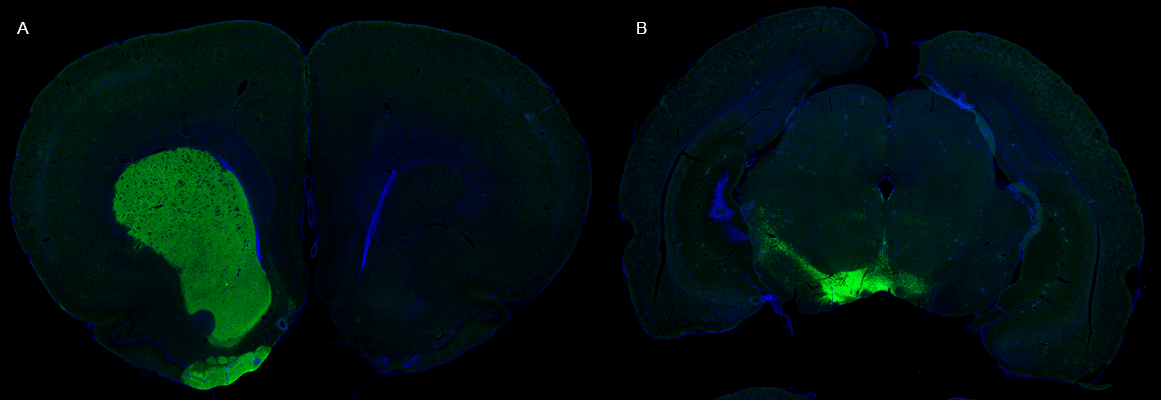

Supplement: Supplementary file 3 — Figure S3. TH staining in the striatum, MFB, and substantia nigra of the 6‐OHDA rats. A. Cross section of the striatum, MFB and substantia nigra; B. TH‐positive neurons in the substantia nigra. [file BTM2-10-e70014-s003.tif]
