# Supplementary material for: Efficacy and safety assessment of homotopical transplantation of iPSCs‐derived midbrain organoids into the substantia nigra of Parkinsonian rats
Source: Bioeng Transl Med. 2025 Mar 27;10(5):e70014. doi: 10.1002/btm2.70014 (PMC12478334; doi:10.1002/btm2.70014)
Supplement: Supplementary file 4 — Table S1. Antibody information. [file BTM2-10-e70014-s002.docx]

**Table S1. Antibody Information.**

| **Primary antibody** | **Species** | **Vendor** | **Catalog#** | **Dilution** | **Assay** |
| --- | --- | --- | --- | --- | --- |
| NESTIN | Mouse | BD bioscience | 611658 | 1:500 | IF |
| NESTIN | Mouse | Millipore | MAB353 | 1:500 | IF |
| SOX2 | Rabbit | Invitrogen | 48-1400 | 1:200 | IF |
| KI67 | Rabbit | Millipore | AB9260 | 1:500 | IF |
| OCT4 | goat | Abcam | AB27985 | 1:200 | IF |
| FOXA2 | Rabbit | Millipore | 07-633 | 1:200-500 | IF |
| FOXA2 | Goat | Santa cruz | Sc-6554 | 1:200-500 | IF |
| EN1 | Mouse | Developmental Studies Hybridoma Bank (DSHB) | 4G11 | 1:20-100 | IF |
| EN1 | Rabbit | Abgent | AP7278A | 1:250 | IF |
| OTX2(biotinylated) | Goat | R&D Systems | AF1979 | 1:50-200 | IF |
| NURR1 | Rabbit | Santa cruz | sc-991 | 1:200 | IF |
| NURR1 | Mouse | Abcam | Ab41917 | 1:200 | IF |
| NURR1 | Goat | R&D Systems | AF2156 | 1:100 | IF |
| TH | Rabbit | Millipore | AB152 | 1:200 | IF, IHC |
| TH | Sheep | Millipore | AB1542 | 1:200 | IF |
| TH | Mouse | ImmunoStar | 22941 | 1:500 | IF |
| TUJ1 | Mouse | Millipore | MAB1637 | 1:500 | IF |
| Girk2 | Rabbit | Alomone Labs | APC-006 | 1:100 | IF |
| Girk2 | Goat | Abcam | Ab65069 | 1:100 | IF |
| Human-specific Synaptophysin 1 | Goat | R&D Systems | AF5555 | 1:100 | IF |
| Serotonin transporter (5-HT) | Rabbit | Abcam | Ab254358 | 1:200 | IF |
| hNA | Mouse | Millipore | MAB1281 | 1:500 | IF |
| hNCAM | Mouse | Santa cruz | Sc-106 | 1:100-200 | IF |
| DARPP32 | Rabbit | Abcam | Ab40801 | 1:200 | IF |
| CHAT | Goat | Millipore | AB144P | 1:200 | IF |
| Vgluta1 | Goat | Abcam | Ab110139 | 1:200 | IF |
| O4 | Mouse | R&D | MAB1326 | 1:200 | IF |
| MBP | Rat | Abcam | Ab7349 | 1:200 | IF |
| GFAP | Rabbit | ZSGB-BIO | ZA-0529 | 1:500 | IF |
